# Weight outcomes audit in 1.3 million adults during their first 3 months’ attendance in a commercial weight management programme

**DOI:** 10.1186/s12889-015-2225-0

**Published:** 2015-09-10

**Authors:** R. James Stubbs, Liam Morris, Carolyn Pallister, Graham Horgan, Jacquie H. Lavin

**Affiliations:** Nutrition and Research Department, Slimming World, Clover Nook Road, Somercotes, Alfreton, Derbyshire DE55 4RF UK; College of Life and Natural Sciences, University of Derby, Kedleston Road, Derby, DE22 1GB UK; Biomathematics and Statistics Scotland, The Rowett Institute of Nutrition and Health, Greenburn road, Aberdeen, AB21 9SB UK

## Abstract

**Background:**

Over sixty percent of adults in the UK are now overweight/obese. Weight management on a national scale requires behavioural and lifestyle solutions that are accessible to large numbers of people. Evidence suggests commercial weight management programmes help people manage their weight but there is little research examining those that pay to attend such programmes rather than being referred by primary care. The objective of this analysis was to evaluate the effectiveness of a UK commercial weight management programme in self-referred, fee-paying participants.

**Methods:**

Electronic weekly weight records were collated for self-referred, fee-paying participants of Slimming World groups joining between January 2010 and April 2012. This analysis reports weight outcomes in 1,356,105 adult, non-pregnant participants during their first 3 months’ attendance. Data were analysed by regression, ANOVA and for binomial outcomes, chi-squared tests using the R statistical program.

**Results:**

Mean (SD) age was 42.3 (13.6) years, height 1.65 m (0.08) and start weight was 88.4 kg (18.8). Mean start BMI was 32.6 kg/m^2^ (6.3 kg/m^2^) and 5 % of participants were men. Mean weight change of all participants was −3.9 kg (3.6), percent weight change −4.4 (3.8), and BMI change was −1.4 kg/m^2^ (1.3). Mean attendance was 7.8 (4.3) sessions in their first 3 months. For participants attending at least 75 % of possible weekly sessions (*n =* 478,772), mean BMI change was −2.5 kg/m^2^ (1.3), weight change −6.8 kg (3.7) and percent weight change −7.5 % (3.5).

Weight loss was greater in men than women absolutely (−6.5 (5.3) kg vs −3.8 (3.4) kg) and as a percentage (5.7 % (4.4) vs 4.3 % (3.7)), respectively. All comparisons were significant (*p <* 0.001). Level of attendance and percent weight loss in the first week of attendance together accounted for 55 % of the variability in weight lost during the study period.

**Conclusions:**

A large-scale commercial lifestyle-based weight management programme had a significant impact on weight loss outcomes over 3 months. Higher levels of attendance led to levels of weight loss known to be associated with significant clinical benefits, which on this scale may have an impact on public health.

## Background

Overweight, obesity and associated diseases are key societal challenges in the developed world. Projected obesity trends and associated health care costs are well documented [1, 2]. Obesity currently accounts for 3–8 % of health costs and 10–13 % of deaths in different parts of Europe [3]. The overall impact on health care costs are estimated to range from €59 billion (direct) €118–236 billion (indirect), because obesity is linked to a range of physical and psychological illnesses [4]. In the United States and United Kingdom costs attributable to obesity and overweight are projected to increase significantly in the future [5–7].

While prevention is preferable to cure, the majority of European adults are already overweight and obese [4], which emphasises the need to provide self management solutions to prevent further weight gain and promote sustained weight loss [8, 9]. Governments are calling for the general population to focus on the proactive prevention of avoidable disease by taking more responsibility for their own health through the adoption of healthier lifestyles, improved diets, increased physical activity and managing their own weight [10, 11].

Obesity prevention and management at the individual level requires support to facilitate, encourage and motivate people to make behaviour changes that will lead to healthier diets, greater participation in physical activity and help manage the stresses that can undermine the adoption of more healthful behaviours [9]. At the level of the general population obesity prevention and management requires solutions that are embedded in the community to encourage sustainable lifestyle changes in larger numbers of people. Thus key public health challenges for obesity prevention and treatment lie in *engaging* the general population to participate in modifying their behaviour, *sustaining* these changes to navigate to a healthier lifestyle in the long-term (i.e. relapse prevention and weight loss maintenance) and *scaling* these approaches across the nation through individual and community involvement. The recent (National Institute of Health and Care Excellence) NICE guidance on managing overweight and obesity in adults recognises the important role that lifestyle weight management programmes can play in the prevention of weight gain, weight loss and prevention of weight regain [12].

There is growing evidence that commercial programmes, which combine behaviour change techniques with the key motivators for changing habits are effective as the first line in helping people adopt healthier dietary and activity patterns, and in providing support to enable them to sustain these changes [13–23]. Much of the evidence-base for commercial programmes in the UK has come from partnership schemes with primary care, where patients can attend the weight management programmes free of charge [13, 17, 18, 20–22, 24].

However, these commercial/primary care partnership schemes represent the minority of participants engaged in commercial weight management provision. There is little published evidence of the performance of programmes on a large scale, outside of partnerships with primary care (i.e. in which participants self-refer and pay a nominal weekly fee), in terms of attendance and weight outcomes (see [14] for an exception). This is an important gap in the public heath evidence base, because many more people attend such programmes as fee-paying participants. It is not known if the results of primary care partnership programmes [13, 17, 18, 20–22, 24] are comparable to or representative of the vast majority of participants who engage in those programmes as fee-paying members. Furthermore, the individual fee-paying route to weight management places minimal cost burdens on health care services.

A recent set of systematic reviews have highlighted the fact while obesity in men is no less prevalent than in women, men are under-represented in lifestyle weight management programmes [25]. There is considerable interest in describing the proportion of men who attend lifestyle weight management programmes, their weight outcomes and programme adherence compared to women with a view to increasing men’s engagement in weight management.

It is generally recognised that the first steps (Tier 1 and 2) in care pathways for obesity in the UK should be behaviour change and lifestyle modification [12, 26]. However it is still not clear whether lifestyle interventions are as effective across a range of BMI categories. We have previously reported rate and extent of weight loss in a primary care/commercial weight management organisation partnership scheme in 34,271 patients referred for 12 weekly sessions. We found that referral to a commercial organisation was as effective for people with high BMIs who may normally be recommended for more intensive and costly secondary or tertiary care, as for those who are moderately overweight [27]. It is not yet clear what the BMI structure of populations who attend such programmes as fee-paying members is, or how their weight outcomes vary as a function of initial BMI.

Finally, two previous analyses have shown that initial weight loss and attendance were key predictors of weight outcomes over 12 and 24 weeks in referral schemes [21, 22]. It is of interest and value to examine if the same factors are key predictors of weight outcomes in the larger population attending as fee-paying members.

The purpose of this analysis was to provide a large-scale service evaluation of a UK based commercial weight management programme in self-referred, fee-paying participants in terms of rate and extent of weight loss, attendance, comparisons of the outcomes for men and women and to briefly assess basic predictors of weight loss in this study population.

## Methods

### The lifestyle weight management programme

The commercial weight management organisation, Slimming World (www.slimmingworld.com), meets the NICE best practice criteria [12] to help adults adopt the lifestyle behaviour changes needed to reduce weight, prevent weight gain and support long-term weight maintenance. The organisation has an extensive community-based infrastructure of over 13,000 support groups held each week across the UK and Ireland, supporting ~800,000 members seeking to manage their weight and to develop healthy eating and lifestyle behaviours. Groups are located in a variety of local venues at different times and days of the week, making the groups widely accessible for members of the community. Around 98 % of participants access the groups through self-referral and pay weekly (£4.95) to attend their chosen group. This is an open programme, with no fixed duration of membership. Participants can join, leave and re-join as they wish for any length of time as support groups are continuously available week-by-week through the year, to maximise attendance and engagement from members of the community.

The programme consist of a multicomponent approach utilising evidence-based behaviour change techniques in the context of group support targeted to individual needs to help members to make healthier food choices and gradual increases in physical activity [9, 28]. Trained facilitator-led group support provides an environment, which avoids criticism, prescriptive control or judgment and facilitates sustainable health related behavior changes. The support system combines individual attention and group participation in a forum where members discuss experiences, identify their own patterns of behavior and, with the support of the group, develop new ways of overcoming barriers to change to support weight loss and maintenance of weight loss.

Evidence-based strategies and actions taken in the programme to promote and sustain weight loss include:

1. Self-regulation through a variety of techniques including (i) weekly recording of body weight, (ii) use of diaries to self-monitor food, activity, feelings and emotions and energy density risk scaling tools to encourage participants to eat more healthily, (iii) individualised motivation and group support for self-monitoring, goal setting, action plans, contingent reinforcement, and pre-planned strategies for relapse events [9, 29–33].
2. Motivational components for dietary and physical activity change involving practices focused on improving intrinsic motivation and self-efficacy for physical activity and healthy eating [33–37].
3. Emotion regulation and stress management components through a non-judgemental, non-stigmatic and de-shaming environment of social support enabling shared experience with an emphasis on the importance of compassion and self-compassion [38–44].

### Data collection

Data was collected for participants attending groups in the UK and Ireland each week through an electronic data capture system and using calibrated digital weighing scales. Participants were weighed in light clothing on scales with a precision of ± 0.23 kg (SECA bespoke model). Accuracy is ensured by calibration against standard weights, during routine service and scales are checked for notable drift weekly in use. At the point of enrolment and where relevant, during their membership, each participant’s gender, date of birth, weight, height, address and health information were recorded by the group consultant, as reported by individual participants and entered onto the electronic system. Each week the participant returned to group, weight was measured and automatically captured on the electronic system. The same assessment procedures were used across all the studied support groups.

This work was conducted as part of Slimming World’s routine data acquisition and monitoring for all participants. Participants are informed of and give written consent to their anonymised data being used for academic research and statistical purposes when they sign the enrolment form as participants of the programme. Data were collected in a live database using specifically designed data capture architecture and stored on a Microsoft Structured Query Language (SQL) server, 2008 r2. Data were collected and stored in line with the Data Protection Act and Information Governance Level 2. This work was therefore not ethically approved or formally categorised as a service evaluation according to the Health Research Authority’s criteria on defining the differences between research, service evaluation, clinical audit, surveillance and usual practice [45]. Nevertheless, data were analysed according to the exact same principles of a service evaluation [45]. Hence existing data were anonymised and analysed as ‘an intervention in use’ only to ask the question “What standard does this service achieve?”

### The dataset

This report analysed data collected from self-referred, fee-paying participants in the Slimming World programme between January 2010 and April 2012 during their first 14 attendances (looking at a possible 13 weekly weight changes). 13 weight changes represent a quarter of the 52 weeks of a year of possible attendance. This resulted in the inclusion of 1,390,285 records where there was a full electronic attendance and weight record for the duration of their membership.

Data were extracted from the company’s secure SQL database, and subjected to a number of checks for outliers, and anomalous data entries. Exclusions were made to the dataset to remove records that were either clearly erroneous or were outside feasible physiological parameters for changes in energy balance that could occur over a given period of time [46–49]. These were weight change of >10 % in the first week of attendance or >5 % in any subsequent individual week (*n =* 2901 (0.20 %)); a weight reduction of >30 % or a weight gain >20 % over the 14 week attendance period (*n =* 2959 (0.21 %)). Data were also excluded on the basis of pregnancy (*n =* 6412 (0.5 % of the total dataset)), breastfeeding (*n =* 6570 (0.5 %)), self-reported history of an eating disorder (*n =* 3076 (0.2 %)), age ≤18 or ≥80, (*n =* 13,960 (1 %)), height >2.1 m or <1.35 m (*n =* 1623 (0.1 %)), start weight >272.7 kg or <36.4 kg (*n =* 196 (0.01 %)), start BMI >90 or <20 (*n =* 1428 (0.1 %)). Some participants were excluded on multiple grounds so the sum of the individual exclusions will exceed the total participants removed. The total number for all of the records removed for the exclusions listed above was 34,180 (2.5 %) resulting in a final dataset of 1,356,105 records.

### Data analysis

From the raw data collected, start body mass index (BMI), end BMI, BMI change, weight change and percent weight change were calculated. The end weight was calculated based on the participants’ last attendance at group during the 14 week period using the Last Observation Carried Forward (LOCF) approach [50]. Data were also imputed using the Baseline Observation Carried Forward approach (BOCF) [51]. Participants were arbitrarily classified as ‘higher attenders’ if they attended ≥75 % (i.e. 11 or more of 14) sessions. Attendance was analysed as both a categorical cut off for descriptive purposes and as a continuous variable for modelling purposes. Subjects achieving categories of weight loss were summarised at the 5 % and 10 % level following the literature relating to the extent of weight loss associated with clinical benefits [52–54] and Department of Health recommendations for evaluation of lifestyle weight management programmes [55].

The effects of different factors on weight loss were assessed by fitting linear models and examining the significance of fitted terms in these models through regression and Analysis Of Variance (ANOVA). Multiple regressions allowed the size of effects related to specific variables (e.g. attendance, first week’s weight loss) and the impact of other variables (e.g. age, gender, BMI) to be modelled in order to better understand the independent effects of the determinants of weight change. The use of ANOVA enabled us to account for the proportion of the variance in outcomes attributable to specific variables. Where binary outcomes (e.g. achieving 5 % weight loss or not) are reported, the effects of factors were tested by Pearson Chi-squared tests. Associations between weight losses in different weeks were examined by Pearson correlation coefficients. All analysis was performed using the R statistical program (http://www.r-project.org/). Results are expressed as mean (SD) or percent with SE where relevant. Where data are presented for men and women and higher and lower attenders, absolute means are given with statistics adjusted for potential confounding factors such as age and height, in multiple regressions and ANOVA.

### Socioeconomic methods

Where reported postcodes were available (*n =* 1,179,704, 87 % of participants), they were assigned an Index of Multiple Deprivation score [56–59] and quintiled (ranked from 1 = disadvantaged to 5 = advantaged) as an indicator of socioeconomic status.

## Results

### Participants

Characteristics of the 1,356,105 participants (67,139 men; 1,288,966 women (95.0 %)) on enrolling were as follows. The mean (SD) age was 42.3 (13.6) years and start weight was 88.4 kg (18.8). Mean start BMI was 32.6 kg/m^2^ (6.3) and 12.2 % of the participating population had a starting BMI of 40 kg/m^2^ or above. The data in Table 1 indicates the socioeconomic characteristics of the Slimming World population, included in this analysis, reflect the Index of Multiple Deprivation score distributions of the general UK population (within 1–3 % across all 5 quintiles) [56–59]. 35 % of the Slimming World population were in the 2 most deprived quintiles.

**Table 1:** The percentage distribution of slimming world members (*n =* 1,179,704) by indices of multiple deprivation centile, compared to the United Kingdom population

	Q1 (most deprived)	Q2	Q3	Q4	Q5 (least deprived)
Slimming World member (%)	15.9	18.9	19.1	20.2	25.8
UK population (%)	17.2	20.8	18.3	18.9	24.7
Difference between Slimming World membership and UK population (%)	–1.3	–1.9	0.8	1.3	1.1

### Weight change and attendance

#### Last observation carried forward

The characteristics and outcomes for higher attenders and lower attenders are given in Table 2. Mean (SD) weight change of all participants was −3.9 kg (3.6), percent weight change −4.4 %(3.8), BMI after 3 months was 31.2 kg/m^2^ (6.1) and mean BMI change was −1.4 kg/m^2^ (1.3). Mean (SD) attendance was 7.8 (4.3) of a possible 14 sessions. Excluding all subjects who only attended one week and therefore recorded no weight change (*n =* 91,814), mean weight change was −4.2 kg (3.6), percent weight change −4.7 % (3.7), mean BMI change was −1.5 kg/m^2^ (1.3) and mean attendance was 8.2 (4.0). 478,772 (35.3 %) of participants attended at least 75 % of sessions (higher attenders) and 877,333 (64.7 %) were classified as lower attenders. Mean (SD) attendance of higher attenders was 12.5 (1.1) sessions and for lower attenders was 5.1 (2.9) sessions. 37.6 % of the whole population and 75.7 % of the higher attenders lost at least 5 %, 23.1 % of higher attenders and 9.0 % of the whole population lost equal to or more than 10 % of their body weight over the 14 possible sessions.

**Table 2:** The characteristics and weight loss outcomes for higher attenders (*n =* 478,772) and lower attenders (*n =* 877,333). Higher attenders are defined as those who attended ≥75 % of the 14 sessions of the observation period. Lower attenders are those who attended ≤75 % of sessions

	Higher attenders	*n =* 478,772	Lower attenders	*n =* 877,333		
	Average	SD	Average	SD	t-value	*p*-value
Height (m)	1.65	0.08	1.65	0.07	1.04	0.300
Weight (kg)	89.9	19.0	87.6	18.6	62.89	<0.001
Age (years)	44.6	13.8	41.1	13.3	139.44	<0.001
Weight change (kg)	–6.8	3.6	–2.3	2.4	–817.55	<0.001
Weight change (%)	–7.5	3.5	–2.7	2.6	–870.14	<0.001
Attendance (wks)	12.5	1.1	5.1	2.9	1646.67	<0.001
Start BMI (kg/m^2^)	33.2	6.3	32.3	6.2	66.47	<0.001
End BMI (kg/m^2^)	30.7	6.0	31.4	6.2	–73.71	<0.001
BMI change (kg/m^2^)	–2.5	1.3	–0.9	0.9	–839.85	<0.001

#### Comparing last observation and baseline observation carried forward models

Table 3 summarises characteristics and outcomes using LOCF and BOCF models. The percent who had dropped out, and did not to return within the week 14 time window of the study was 0.0, 6.8, 13.4, 20.0, 25.7, 30.9, 35.6, 39.7, 43.4, 46.8, 50.2, 53.7, 58.5, 67.8. Parenthetically, 22.8 % of those who missed at least 25 % of possible sessions (lower attenders) attended at a later date during the subsequent 9 months after the 3-month study (Data not shown).

**Table 3:** The weight loss outcomes for participants using last observation baseline observation carried forward models (*n =* 1,356,105)

	LOCF		BOCF		*P*-value
	Average	SD	Average	SD	
Weight change (kg)	–3.9	3.6	–2.4	3.9	<0.001
Weight change (%)	–4.4	3.8	–2.6	4.1	<0.001
Attendance (wks)	7.8	4.3	7.8	4.3	NS
End BMI (kg/m^2^)	31.2	6.1	31.7	6.2	<0.001
BMI change (kg/m^2^)	1.4	1.3	–0.9	1.4	<0.001

Using the assumption of the BOCF model (i.e. assuming participants regained all their weight if they dropped out at <75 % of total attendance), weight change, percent weight change, and mean BMI change were slightly but significantly lower than using the LOCF model. Attendance statistics were the same for both models as were the percentage losing 5 % and 10 % of their weight.

The remainder of the data analyses are described using the LOCF model (see discussion).

### Higher and lower attenders

Table 2 shows that higher attenders on average were slightly heavier (2.3 kg), older, (3.5 years) had a slightly higher BMI (0.9 kg/m^2^) [both *p <* 0.001] and a similar height on enrolment to lower attenders. Higher attenders lost significantly more weight than lower attenders (6.8 versus 2.3 kg, respectively) *p <* 0.001, during the 14-week period. The same patterns were evident for percent weight loss (7.5 versus 2.7 % respectively, *p <* 0.001) and for change in BMI (−2.5 versus −0.9 kg/m^2^ respectively, *p <* 0.001).

**Fig. 1:**
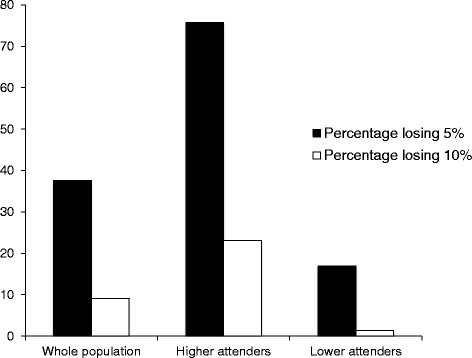
The percentage of the whole population, higher attenders and lower attenders who achieved *≥*5 % and *≥*10 % weight loss by the end of the 14 sessions of the observation period (*n =* 1,356,105)

Figure *1* shows the percentage of the whole population, higher attenders and lower attenders who achieved 5 % and ≥10 % weight loss by week 14. 75.7 % of higher attenders and 16.9 % of lower attenders achieved at least 5 % weight loss (*p* < 0.001). 23.1 % of higher attenders lost at least 10 % of their initial body weight by the end of the referral period compared to 1.3 % of lower attenders (*p* < 0.001).

### Men and women

Table 4 compares the characteristics and weight loss outcomes for men and women. Men were on average taller (0.14 m), older (2 years), heavier (26.2 kg) and had a higher BMI (3.2 kg/m^2^) on enrolment than women (all *p <* 0.001). Men tended to lose more weight than women both absolutely (2.7 kg) and as a percentage (1.4 %) of baseline body weight (both *p <* 0.001). Men attended to a slightly greater degree than women (0.8 sessions) and they had a greater BMI change (−0.7 kg/m^2^) than women over the 14 sessions of the observation period (*p <* 0.001). There was a significant difference in the percentage of men and women classed as higher attenders (42.5 % versus 34.9 %, respectively), (*p <* 0.001).

**Table 4:** The characteristics and weight loss outcomes for men (*n =* 67,139) and women (*n =* 1,288,966)

	Men	*n =* 67,139	Women	*n =* 1,288,966		
	Average	SD	Average	SD	t-value	p-value
Height (m)	1.78	0.07	1.64	0.07	419.61	<0.001
Start weight (kg)	113.3	21.8	87.1	17.7	282.00	<0.001
Age (years)	44.2	13.3	42.2	13.6	20.38	<0.001
Weight change (kg)	–6.5	5.3	–3.8	3.4	–113.16	<0.001
Weight change (%)	–5.7	4.4	–4.3	3.7	–56.18	<0.001
Attendance (wks)	8.5	4.2	7.7	4.3	23.10	<0.001
Start BMI (kg/m^2^)	35.6	6.3	32.4	6.2	95.65	<0.001
BMI at 3 months (kg/m^2^)	33.5	6.1	31.0	6.1	85.91	<0.001
BMI change (kg/m^2^)	–2.1	1.7	–1.4	1.3	–72.78	<0.001

A significantly higher percentage of men than women lost 5 % (51.1 % versus 36.9 %) and 10 % (18.3 % versus 8.6 %, respectively) of their baseline weight by the 14th session (*p <* 0.001).

### Rates of weight change

The average rate of weight change over the 14-week time window, in kg/week for the total population was −0.28 kg/week. ANOVA showed that men lost weight at a significantly faster rate (−0.47 versus −0.27 kg/week or 0.41 versus 0.31 %, respectively) than women, both in absolute and percent terms (*p <* 0.001). Higher attenders lost weight at a significantly faster rate than did lower attenders (−0.48 versus −0.17 kg/week). These patterns were also apparent for percent weight loss −0.53 versus 0.19 %, respectively (both *p <* 0.001).

**Fig. 2:**
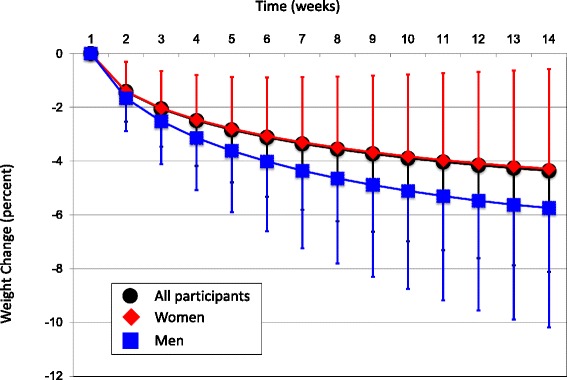
Mean (SD) cumulative weight change for the whole study population (*n =* 1,356,105), women (*n =* 1,288,966) and men (*n =* 67,139) over the 14-sessions of the observation period

Figure 2 shows that the rate of weight loss decreased as the process of weight reduction proceeded from the start to end of the observation period. The slope for the rate of weight loss was steeper during weeks 1 to 7 than weeks 8 to 14 for the whole population (*p <* 0.001, paired *t*-test) and for men compared to women (*p <* 0.001, unpaired *t*-test).

### Weight change in different BMI groups

39.8 % of participants had a BMI <30 kg/m^2^, 30.8 % between 30–34.9 kg/m^2^, 17.3 % between 35–39.9 kg/m^2^ and 12.2 % had a start BMI ≥40 kg/m^2^. Absolute weight loss increased with increasing BMI category (all *p <* 0.001). Absolute weight changes were −3.1, −3.9, −4.5, and −5.4 kg for the BMI categories <30 kg/m^2^, 30–34.9 kg/m^2^, 35–39.9 kg/m^2^ and ≥40 kg/m^2^, respectively (all *p <* 0.001). However, percent weight change was similar in each BMI category at −4.2, −4.5, −4.5 and −4.4 % for BMI categories <30 kg/m^2^, 30–34.9 kg/m^2^, 35–39.9 kg/m^2^ and ≥40 kg/m^2^, respectively (all *p <* 0.03). The significance of these comparisons reflects a small effect size but a very large sample size.

### Prediction of weight loss

Table 5 gives the regression coefficients for percent weight loss at the end of the 3 month period for age, height, gender, starting BMI, attendances and percent weight lost in the first week, together with t-statistics and probability values. The sample (*n =* 1,174,064) necessarily excludes subjects who only attended for 1 week. Change in weight could not be estimated for these subjects (*n =* 182,041). The greatest predictors of percent weight loss were gender (men losing more than women), number of attendances and percent weight lost in the first week (Table 5). Height and start BMI had considerably smaller impacts on percent weight lost at 3 months although due to the very large sample size factors for height and BMI were significant at *p <* 0.001. Table 6 shows the accumulated Analysis Of Variance for the predictors of weight loss, giving the factors, percent of variance explained, F-ratios and probability statistics for main effects. Age, height, gender and starting BMI all explained a very small amount of the variance in percent weight lost. In the case of gender it should be borne in mind that men accounted for 5 % of the sample. Attendance accounted for 44 % of the total variance in percent weight lost at the end of the 3-month period. Percent weight lost during the first week of attendance was also an important predictor of total weight lost, accounting for 11.3 % of the variance. 43.4 % of the variance in weight lost was unaccounted for by this model.

**Table 5:** Regression coefficients for percent weight loss at the end of the 3 month period for age, height, gender, starting BMI, attendances and percent weight lost in the first week, together with t-statistics and probability values (*n =* 1,174,064)

	Estimate	S.E.	t (1,174064)	p-value
Constant	–4.036	0.059	–68.28	<0.001
Age (years)	0.000	0.000	1.03	=0.301
Height (m)	0.023	0.000	67.45	<0.001
Gender (male)	–0.593	0.012	–51.23	<0.001
Start BMI (kg/m^2^)	0.072	0.000	191.25	<0.001
Attendances (wks)	–0.571	0.001	–981.73	<0.001
Weight lost in week 1 (%)	0.627	0.001	552.59	<0.001

**Table 6:** The accumulated analysis of variance for the predictors of weight loss, giving the factors, percent of variance explained, F-ratios and probability statistics for main effects (*n =* 1,174,064)

Change	d.f.	% variance	F-ratio	p-value
Age (years)	1	0.50	13543.70	<0.001
Gender	1	0.79	21272.4	<0.001
Height (m)	1	0.01	312.5	<0.001
Start BMI (kg/m^2^)	1	0.03	802.60	<0.001
Attendances (weeks)	1	43.97	1189414.90	<0.001
Weight lost in week 1 (%)	1	11.29	305358.90	<0.001
Residual	1,174,058	43.41		
Total	1,174,064			

Thus, considering that men accounted for only 5 % of the sample, the two key predictors of percent weight loss by the end of the 3 month period were attendance and percent weight lost in the first week. However, week 1 weight loss was also related to attendance, since adding week 1 weight loss into the stepwise model first increased the percent variance explained by the first week’s weight loss to 19.6 % and decreased the percent of the variance due to attendance to 35.6 %. Since week 1 loss is included in the total loss, some correlation is inevitable unless there is complete compensation in later weeks. Therefore, week 1 loss is positively correlated with subsequent loss up to 3 months. Those who lost more weight in week 1 tended to attend longer. Both models explained the same total amounts of variance in percent weight lost.

## Discussion

About half of all Western adults have attempted weight loss in the last 12 months, the majority without success [60]. At the present time non-commercial behaviour change solutions to weight management are limited in terms of resource, infrastructure and scale. This constrains the ability of health care systems to offer comprehensive weight management solutions on a population level. There is now a growing evidence base documenting how effective commercial behaviour change programmes can be in helping people control their weight [13, 14, 16–23]. Most of this evidence is derived from primary care partnership schemes, often lasting 12 weeks where attendance is free for the participant for the duration of the scheme [13, 15, 18, 19, 22, 24]. In some of these 12-week schemes, weight outcomes have been followed up over 12 months. Some referral schemes have been extended and evaluated over 6 months [21] or over 12 months [17]. One notable study has directly compared commercial and health care-based programmes and found that commercial programmes are more effective in terms of weight outcomes [18]. Further analysis comparing performance of the three main commercial providers and a National Health Service programme in the UK found the commercial providers lead to significantly greater weight loss at 3 and 12 months and that Slimming World led to significantly greater weight loss at 12 months than the reference standard used [20].

Far fewer studies have audited fee-paying services providing community-based weight management services on a large scale e.g. [14]. Slimming World already delivers an effective, national scale infrastructure for weight loss, including networks of local classes, 4000 group facilitators (Consultants), written, online and multi-media resources, eating plans and evidence based behaviour change approaches [22, 27, 28, 61]. The present data set provides an important addition to the UK evidence base for commercial weight management programmes, particularly as this represents the majority of participants accessing such services. To the best of our knowledge this is the first study to report weight outcomes during the first 3 months attendance in over 1 million self-funding participants of a commercial weight management programme. This study shows that fee-paying, general-population behaviour change programmes have the potential to impact on hundreds of thousands of people.

Given the enormous scale of the obesity epidemic, therapeutic approaches to weight management are likely to be most effective if they combine self-management and group management, because traditional one-on-one clinical approaches are too resource intensive and costly to deal with the very large number of affected individuals. One approach to this social problem is through multicomponent behaviour change programmes combining dietary advice, social support structures (group and online support, behaviour change tools, social media) and evidence-based self-management techniques (motivation, emotional support, stress management, goal setting, self-monitoring). In the current study we show how such a programme has a quantitatively significant impact on weight outcomes on a national scale.

### Rate and extent of weight loss

The current audit is the largest examination of the impact of commercial weight management support conducted to date. Weight outcomes were similar to those for primary care partnership schemes published [13, 15, 18, 19, 22, 24]. These studies/evaluations have consistently shown that partnership referral schemes offer an effective service producing ~4–5 % weight loss at 12 weeks and ~5 % at 12 month follow up of 3 month referral [18, 20] or 12 month continuous referral [17]. In the present analysis 37.7 % of the whole population and 75.7 % of the higher attenders lost ≥5 %, which is the current recommended initial target weight loss (within 3–6 months), according to the NICE guidelines and the Department of Health [10, 17]. 23.1 % of higher attenders and 9.0 % of the whole population lost ≥10 % of their body weight over the first 3 months. It is likely that some of those who dropped out early gained more weight than those who dropped out later during the 3-month study period. However, we know that 22.8 % of those who missed at least 25 % of possible sessions (lower attenders) attended at a later date during the subsequent 9 months after the 3 month study window and hence re-engaged with the weight management programme (data not included in this analysis). Modelling the dynamics of participant flow through such open group systems and the impact on longer-term weight outcomes is the subject of future publications. These considerations are encouraging from the perspective of engaging the public in self-management of their weight and health, since most adults tend to gain small amounts of weight over this time period [62–64].

Average start BMI of this population (32.6 kg/m^2^) was in the range that would be recommended for weight management interventions in the UK [17] and was lower than that recorded in our primary care referral schemes. This is primarily due to the fact that this population was characterised by fewer participants with a start BMI ≥40 kg/m^2^, (12.2 %) compared to our referral schemes where 25.4 % had a BMI >40 kg/m^2^. Sixty percent of this population had a BMI over 30 kg/m^2^ suggesting that lifestyle interventions can work on a large scale in populations who are overweight and obese at least over a period of 3 months. The analysis of weight outcomes by BMI groups suggested that lifestyle interventions can be as effective in individuals with higher BMIs as well as for those who are moderately overweight during their first 3 months attendance which is in accordance with previous analysis [27].

### Length of attendance

The data presented in this study supports several analyses of commercial weight management programmes that now suggest the importance of participants attending or complying with their weight loss programme. In all outcomes, higher attenders fared far better than lower attenders. As previously reported, trajectories of weight loss for the higher and lower attender groups were curvilinear, which is typical for obesity treatments in general [65–67]. Weight loss begins more rapidly and starts to slow as weight loss proceeds [9]. A number of studies now suggest that attendance or degree of engagement is a major correlate of weight lost [14, 17, 18, 21, 22, 68]. While the exact mechanisms by which attendance translates into weight loss are not clear it appears that attendance may be related to greater adherence to and use of programme components (i.e. self-regulatory behaviours, behaviour change techniques, support mechanisms) which may well differ for different people. However, it is important to note that this particular analysis cannot identify what specific components are effective, as they were not measured. Attendance appears to be an index of engagement with the multiple components of behaviour change-based weight management programmes, which is related to rate and extent of weight loss. Further studies are needed to assess which methods most encourage engagement in weight management programmes and hence weight outcomes.

### Men and women

As reported in previous publications, absolute and percent weight loss and attendance was higher in men compared to women [21, 22]. While they represent only a small minority of participants in this and other commercial weight management programmes, those men who did engage tended to attend more and lose more weight than women. These data are consistent with the recent NIHR systematic reviews of and integrated report on the quantitative, qualitative and economic evidence base for the management of obesity in men summarising the current knowledge base on how men engage with and respond to weight management services [25]. The present study and these systematic reviews suggest that men are under-represented in lifestyle weight management programmes and that methods to engage more men in these services may not yet be optimised. The current data add insight to the NIHR reviews by suggesting that those men who do engage, attend more and have greater percentage weight loss than women in programmes incorporating dietary change, physical activity components and behaviour change techniques. However, better weight outcomes in men who do engage compared to female participants could be a selection effect associated with other factors such as motivation to engage due to identified health risk and prompting by a health professional [25]. Mechanisms and components of lifestyle weight management programmes are likely to be efficacious for men [25], but further work needs to be undertaken in understanding how to best engage more men in such programmes and how to individualise and personalise those components to their specific needs. It may be thought that because men tend to have a higher (both absolute and percentage) fat free mass than women (and hence higher energy requirements) for a given BMI [69] that they are likely to respond more favourably to attempts at weight reduction. However, higher energy requirements in men lead to higher *ad libitum* energy intakes for a given BMI [70] and we do not consider that the physiology of body composition and energy metabolism of men is the primary factor underlying differences in the responses of men and women to lifestyle-based weight management programmes [46–49]. At present there is insufficient evidence to clearly describe why some men are less likely to engage in lifestyle weight management programmes [25].

### Impact compared to other approaches to weight management

The present results show a mean weight loss of 4.4 % (7.5 % for higher attenders) in the first 3 months of attendance. These data compare very favourably to pharmacological treatments for obesity [71–73] and other commercial and non-commercial weight management programmes [13, 17, 18, 74]. For primary care partnership schemes the average weekly net cost of referral to this organisation equates to £4.13 per week, which compares very favourably to the equivalent prescription costs for obesity drugs [22]. However, the outcomes of the present study incur no net cost to health care providers because this is a paid-for service funded by a participant (weekly fee of £4.95). The results of this study also show that clinically effective weight outcomes can be initiated in the general population on a massive scale. The IMD data demonstrates participants accessing the programme are broadly representative of the general population in terms of socio-economic distribution. It can therefore be reasonably concluded that this model for provision of weight management solutions places minimal burden on health care resources while facilitating effective engagement of the general population in self-management of their weight, health and disease prevention. The scale of the results presented in this paper could have significant potential for far-ranging economic and social impact, helping to alleviate the growing economic burden of treating non-communicable chronic disease associated with obesity [75]. Individually small but population wide reductions in obesity prevalence would have a major impact on related co-morbidities, public health, and health care costs [12]. By supporting individuals in the self-management of their weight, these data show how commercial weight management programmes have the potential to help decrease the prevalence of obesity and incidence of its costly co-morbidities.

### Prediction of weight loss

We have conducted multiple regression analysis on this and two other data sets [21, 22]. These analyses show that initial BMI, absolute weight and age were not strong predictors of weight outcomes. Gender showed a strong regression co-efficient for weight outcomes but did not explain much of the variance. The lower power explained for gender variance could probably (although not certainly) be due to the small percentage of men in the study population. Percent weight lost in the first week and attendance were the greatest predictors of weight loss. These effects has been found in other studies [76–78]. Attendance is discussed above. The relationship between percent weight lost in the first week and end weight loss is less clear. It may be that those who lose a greater percent of baseline weight in the first week are more motivated (either before they attend or as a consequence of their experiences in the first week), they may have familiarised themselves with the eating and activity programme to a greater extent, are simply on a consistent trajectory of greater weight loss or a combination of these variables. What is known is that certain behaviour change techniques are associated with better weight outcomes [29, 30, 74]. Evidence also suggests that greater engagement with components of commercial programmes that promote these behaviour changes is associated with better weight outcomes [68, 77–80]. Improving personalisation of multicomponent programmes to better match behaviour change techniques to the requirements of specific individuals is likely to further improve engagement and weight outcomes. This should be a research priority for implementing weight management solutions in the general population and may improve the capacity of models to predict weight outcomes for different groups of people.

### Imputation of missing data

As in our previous analyses [21, 22] most of the data are presented using the assumptions of the LOCF model to impute missing data. For reference we have also summarised outcomes with the BOCF model. This produced similar outcomes to those seen on other assessments of weight management programmes using different ways to impute missing data e.g. [18]. In the current analysis we argue that LOCF is the most appropriate form of data imputation because BOCF assumptions could assign unrealistic weight regain to participants whose weight loss close to the end of the period is known. In the present analysis attendance was modeled both as a cut off (≥ and ≤75 %), for reference to previous published work [21, 22, 27] and as a continuous variable in multiple regression and ANOVA to model the impact of degree of attendance on weight outcomes.

### Strengths and limitations of the study

Limitations to this study include the absence of a control group and the fact that results were based upon those people who joined a group, rather than intention to treat. Those who join cannot be considered a random sample of the overweight population. The time window was limited to 3 months and the effects of dropout and subsequent re-engagement with the programme were not analysed in this paper. There were no comparable and consistent in-house options available to provide a control group, although commercial-primary care partnerships published by this and other organisations provide a point of reference. The purpose of the study was not to compare the efficacy of this commercial weight management organisation’s programme with other treatments. It was to audit the effectiveness of a population scale, paid for weight management service, in terms of rate and extent of weight loss and attrition rates. Additional studies examining longer-term weight trajectories over 1–2 years, dynamics of engagement, health economics outcomes and the impact of social deprivation on weight outcomes are underway. Key strengths were that the audit assessed the effectiveness of the programme as it runs in real life, the very large sample size and the fact that the subjects were real consumers aiming to control their weight in their everyday lives.

## Conclusions

In the present audit mean weight loss was 3.9 kg, 37.7 % of the whole population and 75.7 % of the higher attenders lost ≥5 % during the first 3 months of their engagement in a commercial weight management programme. Twenty three percent of higher attenders and 9.0 % of the whole population lost ≥10 % of their body weight. These data have confirmed that there are two simple basic predictors (attendance and weight loss in the first week) of weight loss that together account for 55 % of the variability in weight lost during the study period, which would be valuable indicators of the likelihood a person will lose ~5 % of their initial body weight during that time. Weight management programmes should therefore work to enhance initial weight loss and attendance because regardless of age or starting weight, if a person is able to attend 11 or more sessions in a 3 month period and is supported and encouraged to achieve good weight losses in their first week, they are likely to be successful in beginning their weight loss journey. This model for provision of weight management solutions places minimal burden on health care resources while facilitating effective engagement of the general population in self-management of their weight, health and disease prevention. The scale of these data could have significant impact. These data show that by supporting citizens in the self-management of their weight, commercial weight management programmes have the potential to help decrease the prevalence of obesity and incidence of its costly co-morbidities, improving citizen health and helping to reduce the burden on national health care systems.

## Abbreviations

SD: Standard Deviation
BMI: Body Mass Index
ANOVA: Analysis of variance
NICE: National institute of Health and Clinical Excellence
US: United States
UK: United Kingdom
SQL: Structured Query Language
LOCF: Last Observation Carried Forward
BOCF: Baseline Observation Carried Forward
NHS: National Health Service
